# Human exonuclease 1 (EXO1) activity characterization and its function on flap structures

**DOI:** 10.1042/BSR20150058

**Published:** 2015-06-11

**Authors:** Guido Keijzers, Vilhelm A. Bohr, Lene Juel Rasmussen

**Affiliations:** * Center for Healthy Aging, Department of Cellular and Molecular Medicine, University of Copenhagen, Denmark; † Laboratory of Molecular Gerontology, National Institute on Aging, U.S.A.

**Keywords:** double stranded breaks, EXO1, flap activity, strand displacement, threading, tracking

## Abstract

Human exonuclease 1 (EXO1) is involved in multiple DNA metabolism processes, including DNA repair and replication. Most of the fundamental roles of EXO1 have been described in yeast. Here, we report a biochemical characterization of human full-length EXO1. Prior to assay EXO1 on different DNA flap structures, we determined factors essential for the thermodynamic stability of EXO1. We show that enzymatic activity and stability of EXO1 on DNA is modulated by temperature. By characterization of EXO1 flap activity using various DNA flap substrates, we show that EXO1 has a strong capacity for degrading double stranded DNA and has a modest endonuclease or 5′ flap activity. Furthermore, we report novel mechanistic insights into the processing of flap structures, showing that EXO1 preferentially cleaves one nucleotide inwards in a double stranded region of a forked and nicked DNA flap substrates, suggesting a possible role of EXO1 in strand displacement.

## Abstract (precis)

We report biochemical characterization of human full-length EXO1 including thermodynamic stability and flap activity on DNA flap structures. Our results reveal novel mechanistic insights into the processing of flap structures and a possible role of EXO1 in strand displacement.

## INTRODUCTION

Human exonuclease 1 (EXO1) has been implicated in many different DNA metabolic processes, including DNA mismatch repair (MMR), micro-mediated end-joining, homologous recombination (HR), and replication [1–6]. Human EXO1 belongs to a family of eukaryotic nucleases, Rad2/XPG, which also include FEN1 and GEN1 [7,8]. The Rad2/XPG family is conserved in the nuclease domain through species from phage to human. The *EXO1* gene product exhibits both 5′ exonuclease and 5′ flap activity [7,9]. Additionally, EXO1 contains an intrinsic 5′ RNase H activity [8,10]. Human EXO1 has a high affinity for processing double stranded DNA (dsDNA), nicks, gaps, pseudo Y structures and can resolve Holliday junctions using its inherit flap activity [9,11]. Human EXO1 is implicated in MMR and contain conserved binding domains interacting directly with MLH1 and MSH2 [5,6,12,13]. EXO1 nucleolytic activity is positively stimulated by PCNA, MutSα (MSH2/MSH6 complex), 14-3-3, MRN and 9-1-1 complex [11,14–17]. Studies of *Saccharomyces cerevisiae* (*S. cerevisiae*) imply that EXO1 may function as a backup for Rad27 (FEN1) in primer removal and Okazaki fragment maturation during DNA replication [18,19]. FEN1 can process short 5′ overhangs efficiently by tracking, whereas long 5′ overhangs (>20–30 nt) are processed by threading in which case FEN1 requires the assistance of DNA2 [20,21]. Pol δ exonuclease activity can remove 1 and 2 nt 5′ flaps efficiently even in the absence of FEN1 or in the presence of inactive FEN1, but the reaction is limited to this flap size [22]. *In vitro* studies suggest that EXO1 forms complexes with the RecQ helicases RECQL1, BLM and WRN, which stimulate its nuclease activity [23–27]. During resection, the EXO1 activity is regulated by RPA, Ku70/80 or CtIP [28–31].

**Figure 1:**
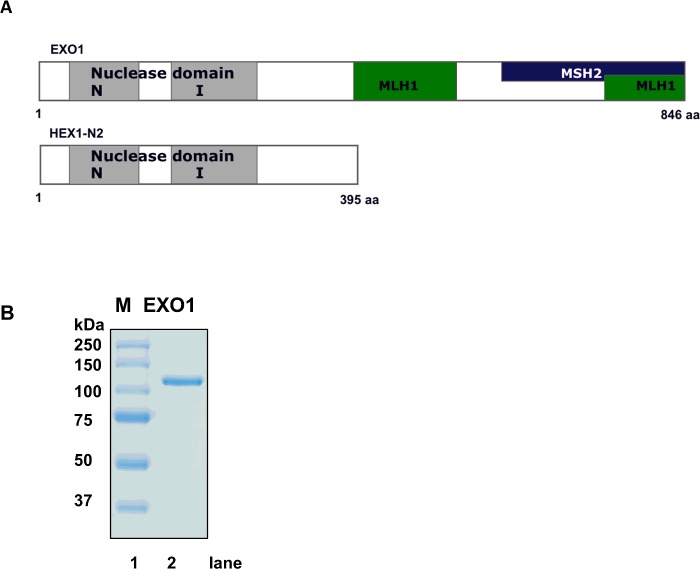
Overview of EXO1 variants and purified full-length EXO1 (**A**) Schematic overview of full-length EXO1 and HEX-N2. Binding domains to MLH1, and MSH2 indicated at the COOH-terminus of full-length EXO1. In both full-length EXO1 and HEX-N2, the nuclease domain contain two domains, N and I domain, separated by a spacer. (**B**) SDS/PAGE analysis of purified EXO1. SDS/PAGE (8% gel). Lane 1=molecular weight marker (size in kDa on the left). Lane 2=purified EXO1.

**Table 1:** DNA sequences of substrates used in the experiments

*dsDNA 42 bp*	
GK142	5′-GACAGGATCCGGGCTAGCATCTTCATACATAGTTGTCACTGG
GK143	5′-CCAGTGACAACTATGTATGAAGATGCTAGCCCGGATCCTGTC
*5*′-*flap substrate, 21 bp duplex dsDNA*	
GK145	5′-ATTGGTTATTTACCGAGCTCGAATTCACTGG
GK135	5′-CCAGTGAATTCGAGCTCGGTACCCGCTAGCGGGGATCCTCTA
*5*′-*flap substrate, 21 bp duplex dsDNA blocked by 5*′-*biotin*	
GK145	5′-ATTGGTTATTTACCGAGCTCGAATTCACTGG
GK144	5′-biotin-CCAGTGAATTCGAGCTCGGTACCCGCTAGCGGGGATCCTCTA
*5*′-*flap substrate, 16 bp duplex dsDNA*	
GK230	5′-ATTGGTTATTATTTCAGCTCGAATTCACTGG
GK135	5′-CCAGTGAATTCGAGCTCGGTACCCGCTAGCGGGGATCCTCTA
*5*′-*flap substrate, 12 bp duplex dsDNA*	
GK229	5′-ATTGGTTATTATTTTTTTACGAATTCACTGG
GK135	5′-CCAGTGAATTCGAGCTCGGTACCCGCTAGCGGGGATCCTCTA
*5*′-*flap substrate, 8 bp duplex dsDNA*	
GK228	5′-ATTGGTTATTATTTTTTTATTTTTTCACTGG
GK135	5′-CCAGTGAATTCGAGCTCGGTACCCGCTAGCGGGGATCCTCTA
*5*′-*flap substrate, forked hairpin substrate*	
GK232	5′-TTTTTTTTTGAGAGGATCCAGCTTTTTTGCTGGATCCTCTCCGCCAGGGT
*1–5, 9 and 10 nt overhang, forked hairpin substrate*	
GK251	5′-TGAGAGGATCCAGCTTTTTTGCTGGATCCTCTCCGCCAGG
GK252	5′-TTGAGAGGATCCAGCTTTTTTGCTGGATCCTCTCCGCCAGG
GK255	5′-TTTGAGAGGATCCAGCTTTTTTGCTGGATCCTCTCCGCCAGG
GK257	5′-TTTTGAGAGGATCCAGCTTTTTTGCTGGATCCTCTCCGCCAGG
GK253	5′-TTTTTGAGAGGATCCAGCTTTTTTGCTGGATCCTCTCCGCCAGG
GK246	5′-TTTTTTTTTGAGAGGATCCAGCTTTTTTGCTGGATCCTCTCCGCCAGG
GK247	5′-TTTTTTTTTTGAGAGGATCCAGCTTTTTTGCTGGATCCTCTCCGCCAGG
*1, 5 and 10 nt overhang, closed nick substrate*	
GK257	5′-TGGTACCGAGTTCTCGGTACCGCCGCTAGCTTGCTAGCGGC
GK258	5′-TTTTTGGTACCGAGTTCTCGGTACCGCCGCTAGCTTGCTAGCGGC
GK259	5′-TTTTTTTTTTGGTACCGAGTTCTCGGTACCGCCGCTAGCTTGCTAGCGGC
*1, 5 and 10 nt overhang, gap substrate*	
GK260	5′-TGGTACCGAGTTCTCGGTACCGCCGCTAGCTTGCTAGCGG
GK261	5′-TTTTTGGTACCGAGTTCTCGGTACCGCCGCTAGCTTGCTAGCGG
GK262	5′-TTTTTTTTTTGGTACCGAGTTCTCGGTACCGCCGCTAGCTTGCTAGCGG
*1, 5 and 10 nt overhang, closed gap substrate*	
GK249	5′-TTTTTTTTTTTTTTTTTTTTGAGAGGATCCAGCTTTTTTGCTGGATCCTCTCCGCCAGG
GK250	5′-TTTTTTTTTTTTTTTTTTTTGAGAGGATCCAGCTTTTTTGCTGGATCCTCTCCGCCAGG
GK254	5′-TTTTTT(TEG-biotin)TTTTTTTTTTTTTGAGAGGATCCAGCTTTTTTGCTGGATCCTCTCCGCC
*Size markers, 10 bp and 12 bp*	
GK245	5′-TTCACCGGTA
GK196	5′-AATTCACCGGTA

The EXO1 nuclease region contains two subdomains, the NH_2_-terminal (N) domain and the internal (I) domain separated by a spacer region (Figure 1A). The N-domain facilitates binding of EXO1 to the DNA [8], while the I-domain exhibits multiple cysteine and glutamate residues that are required for the binding of Mg^2+^ [8]. The loading of two Mg^2+^ on to the I-domain facilitates the binding of EXO1 to the phosphate groups on the DNA molecules prior to DNA hydrolysis [8].

So far, most biochemical studies have been focused on the NH_2_-terminal region of EXO1, also named HEX-N2, which represents 395 of the 846 amino acids full-length EXO1 (Figure 1A) [7–9]. The HEX-N2 variant, which contains an active exonuclease domain, has been purified from bacteria and has recently been characterized by crystallography [8]. Full-length EXO1 has only been partially characterized and this has been limited to study its biochemical activity at various salt and pH conditions [32]. Recently, it was shown that characterization of the full-length EXO1 is essential in order to understand the biochemical mechanisms underlying exonucleolytic activity of the protein [32–35]. A mutated form of EXO1, EXO1-Glu-109-Lys, was biochemically characterized and reported to be compromised in nuclease activity; however, the mutated amino acid was not located within the conserved region [7,35,36]. In a recent study, using the EXO1-Glu-109-Lys knockin mouse, only minor differences in MMR activity was found comparing with wild type [33].

Here, we report novel findings on the characterization of full-length EXO1. We focus on thermodynamic factors such as temperature and salt conditions, which influence protein stability. We show that EXO1 is more active on DNA at temperatures below 37°C and this manifests as an increase in nuclease activity. We also studied the specificity of the endonuclease activity of EXO1 by using specific DNA substrates and report novel mechanistic insights into the processing of flap structures. We show that EXO1 preferentially cleaves one nucleotide inwards in a double stranded region of forked and nicked DNA flap substrates, suggesting a possible role of EXO1 in strand displacement.

## EXPERIMENTAL PROCEDURES

### Protein purification

Expression and purification of human EXO1 in the Bac-to-Bac expression system was previously described [15]. Purity of EXO1 has been analysed on an SDS/PAGE (8% gel) and estimated to be 95%.

### Protein assaying

Oligos (TAG Copenhagen) used as DNA substrate for the different experiments are shown in Table 1. Labelling of 5′-labelling of the DNA oligos and the EXO1 assay were previously described [15]. Terminaldexoytransferase (TdT) (Fermentas) was used for 3′-labelling of oligo GK142, in presence of α-[^32^P]-dGTP (PerkinElmer) and 5× TdT buffer and incubated for 15 min. Non-incorporated α-[^32^P]-dGTP was removed by the illustra MicroSpin column G-25 (GE-Healthcare). Radiolabelled GK142 was annealed with DNA oligo GK143 in the presence of 100 mM KCl. Purified EXO1 was assayed under similar conditions as 5′-labelled oligos as previous described [15]. Oligos GK145, GK230, GK229, GK228 used for generation of DNA flap substrates were 5′-radioactive labelled by PNK as described above. Annealed by heating to 93°C and cooled to room temperature in the presence of 100 mM KCl and complementary strand (either GK144 or GK135) (see Table 1). Hairpin oligo substrates GK232, GK251, GK252, GK253, GK246, GK247 and circular substrates GK257, GK258, GK259, GK260, GK261, GK262, GK249, GK250 and GK254 were 5′-labelled. The oligos were heated to 93°C and cooled to room temperature and annealed by self-annealing to form the hairpin or loop structure in presence of 100 mM KCl. The EXO1 exo- and endonuclease reactions were assayed in reaction mixture of total volume of 10 μl containing 0.06 pmol radioactive labelled substrate and EXO1 protein (between 0.5 and 3 pmol) in 20 mM Hepes buffer containing 100 mM KCl, 0.5 mM DTT, 5 mM MgCl_2_, 0.05% Triton X-100, 5% glycerol and 100 μg/ml BSA. The amount of KCl was varied during testing of exonuclease activity in different salt conditions (in range of 50–250 mM KCl) (Supplementary Figure S1). Reactions were incubated for 30 min at 37°C and stopped by addition of 3.5 μl formamide buffer and heated to 90°C for 5 min. Different incubation temperatures were used in Figures 2(C) and 2(D) (25°C and 30°C). The reactions were separated on a 12% denaturing polyacrylamide gel and reaction products visualized by phosphorimaging. Imaged product and excised product ratios were determined using ImageQuant 5.2 (Molecular Dynamics). In Figures 4(C) and 6(A) the samples were treated post reaction with 1% SDS and 2 μl protease K (10 mg/ml stock) for 30 min at 55°C, to remove streptavidin followed by a denaturing step by addition of 6 μl formamide buffer and heating to 90°C followed by gel electrophoresis as described above. Supplementary Figure S2 shows SDS/PAGE images that were analysed and quantified by use of ImageJ (version 1.49p) software.

**Figure 2:**
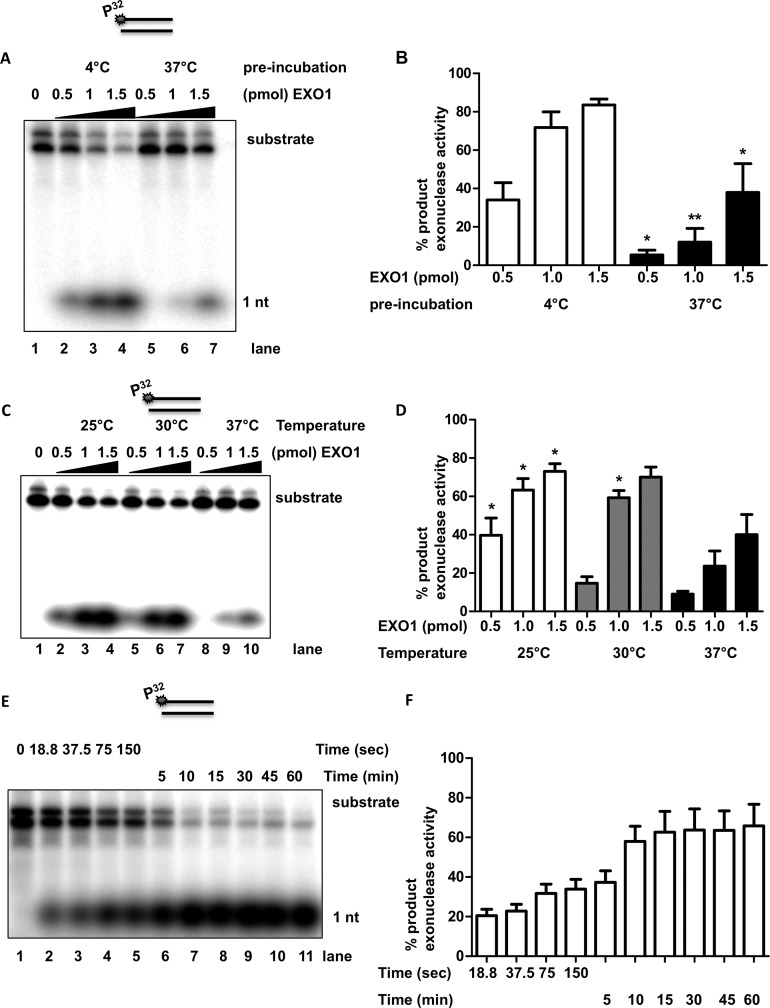
Temperature modulates EXO1 activity (**A**) Heat inactivation of EXO1 at 37°C. Representative gel of three independent biological replicas showing nuclease activities on a 5′ radioactive labelled 42-mer dsDNA GK142/GK143 (star denotes 5′-radiolabelling site), by incubation with recombinant purified EXO1. Lane 1 is substrate control, no EXO1 enzyme was added. Lanes 2–4 and lanes 5–7, both contain an increased amount of EXO1 0.5–1.5 pmol. Lanes 2–4 were pre-incubated at 4°C for 30 min prior the setup of the EXO1 assay (37°C for 30 min), acting as control for full activity. Lanes 5–7 were pre-heated for 30 min at 37°C, prior the EXO1 assay (37°C for 30 min). (**B**) Student *t*-test of EXO1 nuclease activity at 4°C and 37°C. Graph demonstrates that pre-incubation of EXO1 at 37°C results in a significant decrease in exonuclease activity, relative to incubation at 4°C (*P<0.05, **P<0.01), by comparing exonuclease product per EXO1 concentration. (**C**) Lower temperature increases EXO1 activity. Representative gel of four independent biological experiments showing that nuclease activities on a 5′-radioactive labelled 42-mer dsDNA (GK142/GK143) (star denotes 5′-radiolabelling site), by incubation with recombinant purified EXO1. Lane 1 is DNA substrate control, no EXO1 enzyme was added. Lanes 2–10, show EXO1 assay incubated with three different temperatures, respectively 25°C, 30°C, and 37°C, with increased concentration of EXO1 (0.5–1.5 pmol). (**D**) Student *t*-test of EXO1 nuclease activity at 25°C and 30°C versus 37°C. The EXO1 activity at 25°C was significantly increased in exonuclease activity compared with 37°C (*P<0.05). At 30°C, EXO1 only showed a significant increase in activity at 1 pmol versus at 37°C (*P<0.05). (**E**) Time course of EXO1 activity. Representative gel of three independent biological experiments showing that nuclease activities on a 5′-radioactive labelled 42-mer dsDNA GK142/GK143 (star denotes 5′-radiolabelling site), by incubation at time point, respectively 18.8, 37.5, 75, 150 s, 5, 10, 15, 30, 45, and 60 min with of EXO1 (2 pmol). (**F**) Graph shows analysis of EXO1 nuclease activity at different time points between 18.8 s and 60 min. The Student *t*-test indicated that there is no significant difference (*P*<0.05) between exonuclease product from the time points 10, 15, 30, 45, and 60 min.

### Statistical analysis

Statistical comparisons were determined using GraphPad Prism 5.04 software (La Jolla, CA, USA) and the Student *t*-test (Figures 2B, 2D, 2F and S2B) or one-way analysis of variance with Tukey's multiple-comparison test (Figures 5E, 6B, 7D and S1B) of at least three independent biological experiments. Variance between replicates is indicated by standard error of the mean. *P*-values are indicated in the figure legends. Error bars in the all graphs are indicated by the ±SEM.

## RESULTS AND DISCUSSION

Reports in the literature describing the characterizing of full-length EXO1 protein are still limited. However, the importance of such studies has recently been demonstrated by biochemical studies that followed up on the MMR-deficient phenotype of the EXO1-Glu-109-Lys mouse. These studies revealed that the exonuclease activity of this EXO1 mutant was similar to wild-type protein [32,34]. Along this line, characterization of EXO1 exonuclease activity on various substrates is still incomplete such as EXO1 activity on DNA flap substrates. Recently, it was suggested that NH_2_-terminal tagging of EXO1 inhibited nuclease activity [34]. Therefore, we investigated biochemical and thermodynamic properties of full-length EXO1, without affinity tags, on different DNA substrates. Human EXO1 was purified to near homogeneity (Figure 1B) using a strategy previously described [15]. EXO1 has a molecular weight of 94 kDa, but migrates similar to proteins of about 115 kDa in size in the presences of sodium dodecyl sulfate, as previously observed [37]. Thermodynamic parameters such as pH have been shown to affect the nucleolytic activity of truncated EXO1 and recently also of the full-length EXO1 protein [9,32]. To confirm if full-length EXO1 has similar characteristics as the truncated version HEX-N2, we analysed thermodynamic parameters such as temperature and salt conditions.

### Thermodynamic parameters of EXO1

First, we assayed the salt tolerance of full-length EXO1. It was previously shown that truncated HEX-N2 displayed optimal activity at 50 mM KCl [9]. Recently, it was shown that full-length EXO1 has reduced nuclease activity at 40 mM and 180 mM KCl [32]. We show that full-length EXO1 is tolerant to KCl in the range from 50 mM to 150 mM and thus active under physiological salt conditions (Supplementary Figure S1A, lanes 2–4 and S1B). High concentrations of KCl (200–250 mM) reduced EXO1 nuclease activity (Supplementary Figure S1A, lanes 5–6 and S1B). Further characterization of EXO1 was done in the presence of 100 mM KCl to stabilize the DNA configurations, by reducing thermo-breathing of DNA forming transient 5′ flap at dsDNA.

To examine if the EXO1 protein is thermo-stable, we pre-incubated EXO1 at either 37°C or 4°C for 30 min, followed by an exonuclease assay using GK142/GK143 as dsDNA substrate (Figure 2A) [9]. Pre-incubation of EXO1 at 4°C (Figure 2A, lanes 2–4 and 2B) increased exonuclease activity 2-fold compared with 37°C (Figure 2A, lanes 5–7, and 2B). The reactions were analysed under denaturing condition on 12% acrylamide gel. The substrate migrated, under these conditions, as two bands. The product of the reaction is the 1 nt product. To confirm that EXO1 is thermo-sensitive, we tested EXO1 activity at three different temperatures, 25°C, 30°C and 37°C (Figures 2C and 2D). We observed that EXO1 has approximately a 2-fold increase in enzymatic activity at 25°C and 30°C compared with 37°C (Figure 2C, lanes 2–7 and 2D).

These observations are important for the design of *in vitro* experiments using EXO1 since many assays are performed at room temperature. Next, we investigated the role of reaction incubation time, since we observed that EXO1 activity decreased after 30 min at 37°C. We examined if the EXO1 protein would remain active after 18.8, 37.5, 75, 150 s, 5, 10, 15, 30, 45, and 60 min in reaction (Figures 2E and 2F). We assayed the reaction at 37°C and found that EXO1 degraded 5′ labelled substrates rapidly during the first 10 min. After 15 min there was no increase in degradation of the 5′ labelled DNA substrate; this was observed for several concentrations of EXO1 (data not shown). These data suggest that EXO1 is inactive in reaction mixture after 10 min at 37°C concentration of EXO1 (Figures 2E and 2F). To make sure that this is not related to contamination of proteases in the purified EXO1 fraction we used, we assayed the possible degradation of EXO1 at 37°C after 0, 15, 30, 45, and 60 min (Supplementary Figure S2A). We did not detect any degradation of EXO1 under these conditions (Supplementary Figure S2A and S2B). Thus, our results suggest that the EXO1 protein hydrolyses DNA rapidly in the beginning of the reaction, followed by deactivation of the enzyme.

**Figure 3:**
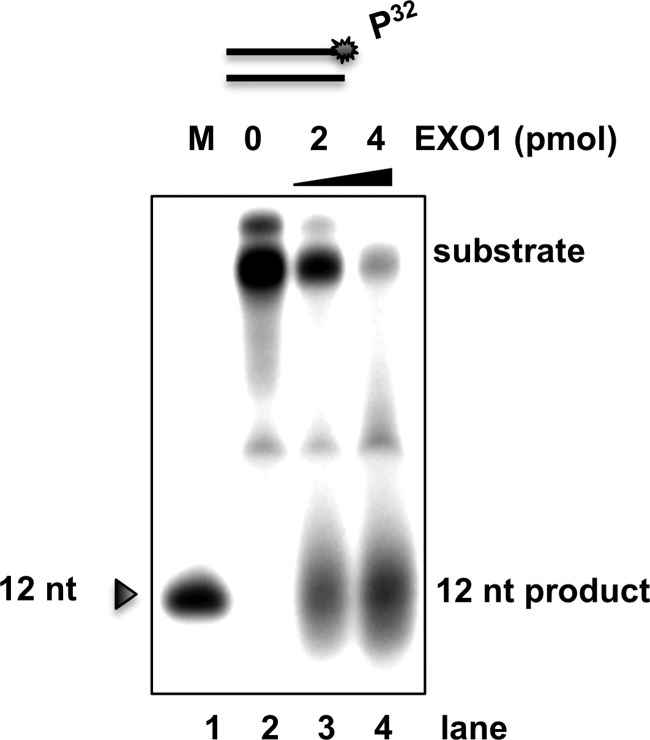
EXO142-mer dsDNA substrate yield in a 12 bp product The gel image showing EXO1 exonuclease activity on a 3′ labelled DNA substrate. Representative gel of three independent biological experiments showing that nuclease activities on a 3′-radioactive labelled 42-mer dsDNA (GK142/GK143) (star denotes 3′-radiolabelling site), by incubation with recombinant purified EXO1. In lane 1, a radioactive labelled marker was used to indicate 12 bp (GK192). In lanes 2–4, dsDNA substrate, 3′ labelled was used. In lane 2, no EXO1 enzyme was added. In lanes 3 and 4, 2 and 4 pmol EXO1 was added, respectively, and the oligo was degraded for 30 min at 30°C.

**Figure 4:**
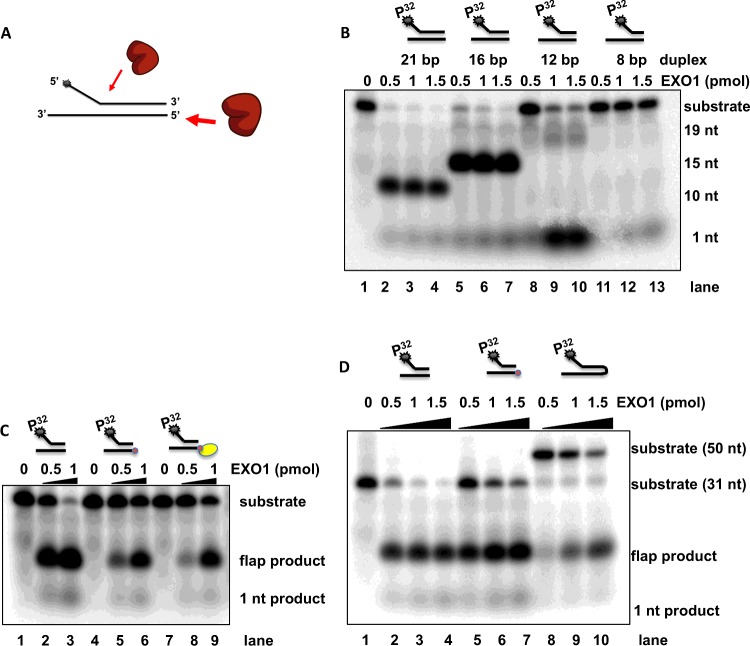
Assaying EXO1 endonuclease activity on different forked substrates (**A**) Schematic overview of EXO1 processing a forked DNA substrate. The classically used forked substrate can be processed by endonuclease activity at the 5′-flap and exonuclease activity at the dsDNA end. EXO1 is shown in brown, red arrow points to the forked substrate sites of action (star denotes 5′-radiolabelling site). (B) The gel image shows EXO1 activity on four flap substrates differing in the length of dsDNA duplex. Representative gel of three independent biological experiments, showing endonuclease activity on a forked substrate 5′-radioactive labelled at flap (star denotes 5′-radiolabelling site). Lane 1 control, no EXO1 enzyme was added. Lanes 2–4 contains the classical flap substrate GK145/GK135 (21 bp duplex), lanes 5–7 contains flap substrate GK230/GK135 (16 bp duplex), lanes 8–11 GK229/GK135 (12 bp duplex), lanes 12–14 (8 bp DNA duplex) with increased concentration of EXO1 (0.5–1.5 pmol). Shortening of the duplex leads to reduced flap activity. (**C**) The gel image shows EXO1 flap activity on three fork substrates. Representative gel of three independent biological experiments showing nuclease activities on three different 5′-radioactive labelled flap substrates (star denotes 5′-radiolabelling site). Lane 1, no EXO1 enzyme was added, substrate control. Lanes 1–3 contain the classical forked DNA substrate GK145/GK135 with increased concentration of EXO1 (0.5–1.5 pmol). In lanes 4–6 the dsDNA end was 5′ blocked with a biotin. In lanes 7–9 the dsDNA end was 5′ blocked with a biotin-streptavidin. Blocking of the 5′ dsDNA end of the fork substrate led to decreased flap activity. (**D**) EXO1 flap activity on two forked and one hairpin DNA substrate. Representative gel of three independent biological experiments showing nuclease activities on a 5′-radioactive labelled at the flap. Lane 1, DNA substrate control, no EXO1 enzyme was added (GK145/GK135). Lanes 2–4 contain forked DNA substrate GK145/GK135, with increasing amount of EXO1 enzymes (0.5–1.5 pmol). Lanes 5–7 contain the GK145/GK144 DNA substrate 5′ biotin blocked at the dsDNA end. Lanes 8–10 contain a hairpin DNA substrate GK232, with increasing amounts of EXO1 enzyme.

**Figure 5:**
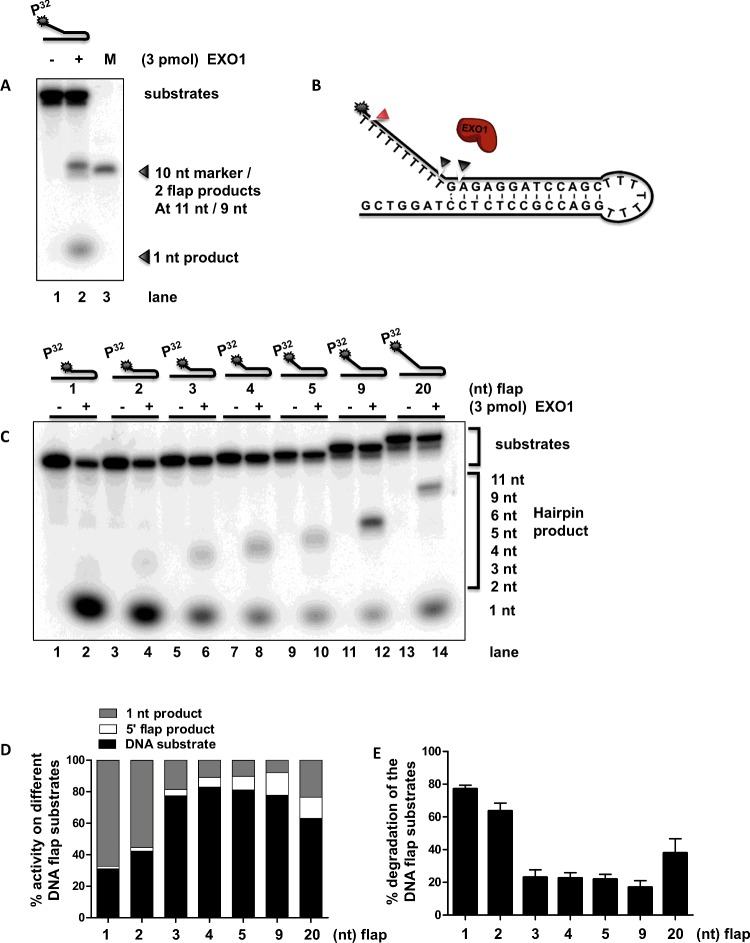
EXO1 activity on a forked DNA substrate (**A**) The gel image shows EXO1 activity on flap substrates. Lanes 1 and 2 contain a 5′ label DNA substrate GK247 in absence and presence of EXO1 (star denotes 5′-radiolabelling site). Lane 3 contains 5′ label 10 nt oligo (GK245). (**B**) Schematic overview of EXO1 processing hairpin forked DNA substrate. The forked substrate with a 10 nt flap is cleaved 1 bp inwards at the dsDNA resulting in 11 nt and smaller product of 9 nt (indicated with grey arrow). The 1 nt cleavage position at the ssDNA is indicated with a red arrow. (**C**) EXO1 process short flaps efficiently. Representative gel of five independent biological repeats of EXO1 showing nuclease activities on 5′-flap DNA substrates in length between 1 and 20 nt 5′ flap (star denotes 5′-radiolabelling site). DNA oligos GK251–GK253 are in range of 1 nt–5 nt 5′ flap and GK246 and GK249 contain respectively 9 nt and 20 nt 5′-flap. The different DNA substrates are setup in absence and presence of EXO1. (**D**) Analysis of the distribution of substrate and degraded flap and 1 nt product. (**E**) Statistical analysis of the degradation of the flap substrates. One-way analysis of variance with Tukey's multiple-comparison test of the EXO1 nuclease activity in salt condition. Percent of EXO1 activity on different 5′-flap DNA substrates. The 1 and 2 nt DNA 5′-flap are both significantly (***P<0.001) more degraded than longer flaps (>2 nt).

**Figure 6:**
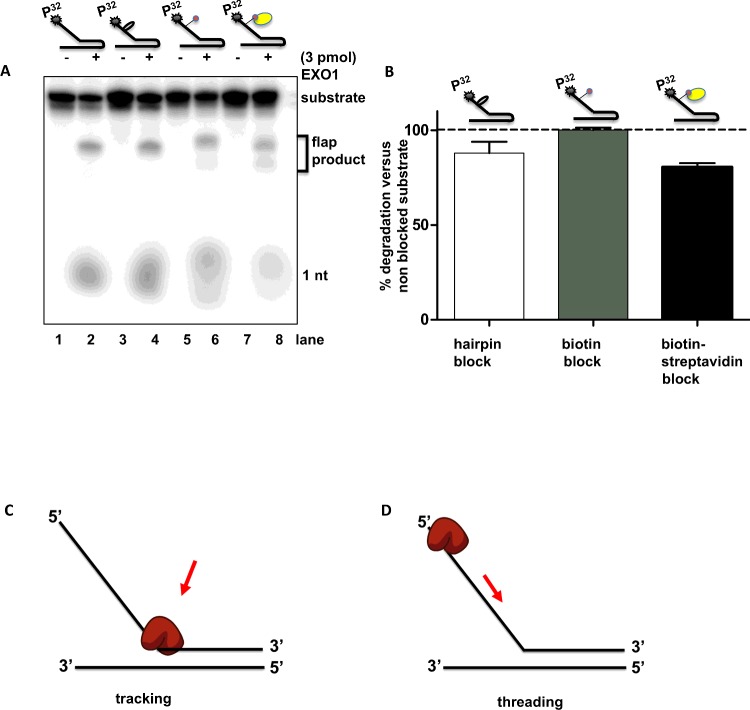
Tracking or threading of EXO1 along the ssDNA flaps to junction (**A**) Representative gel of five independent biological experiments showing that EXO1 detects the 5′ DNA 20-mer flap by tracking and threading. Lanes 1–2 contain substrate GK249, lanes 3–4 substrate GK250, which contain a hairpin loop in the ssDNA (starting at nucleotide position 6 from the 5′ end), and lanes 5–6 substrate GK254 which contains a TEG-biotin, at position 6 nt from 5′ end on the ssDNA and lanes 7–8 substrate GK254 contains in presents of streptavidin. Lanes 2, 4, 6 and 8 are substrates in the presence of EXO1. (**B**) Statistical analysis of degradation of the different DNA conversions. One-way analysis of variance with Tukey's multiple-comparison test. GK250 shows a trend in versus GK249. GK254 and GK249 show a similar degradation. (**C**) A schematic model of tracking, where EXO1 detect the junction of the 5′ flap and cleave directly. (**D**) A schematic model of threading, where EXO1 detects the ssDNA end at the 5′ flap and slide of the ssDNA to the junction of the 5′ flap and cleave.

**Figure 7:**
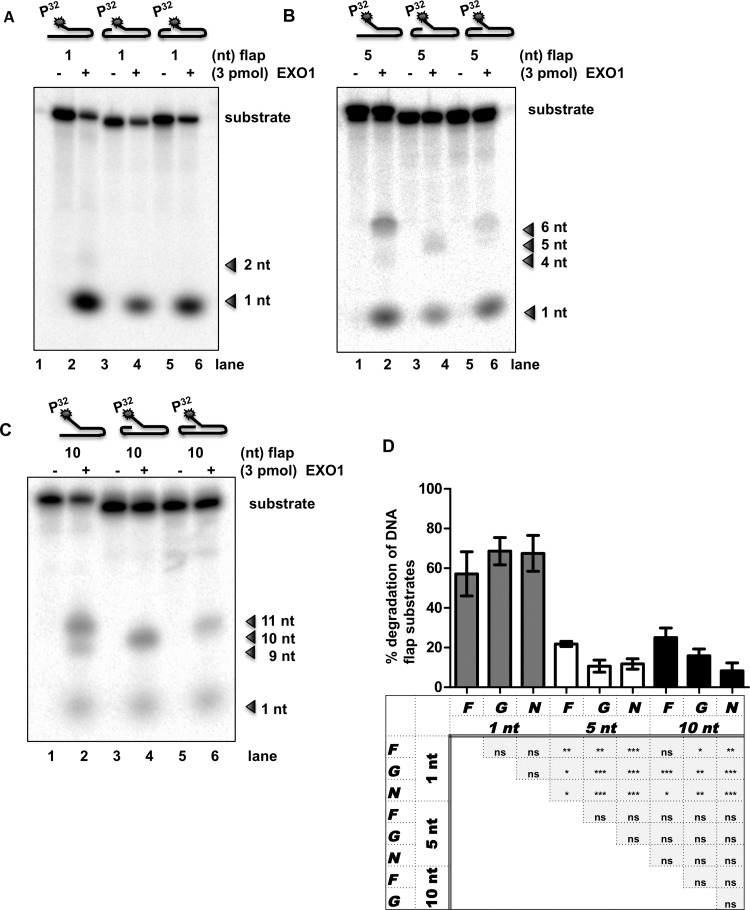
Fork, gap and nick DNA configurations are differently processed by EXO1 (**A**), (**B**) and (**C**) Representative gels of at least three independent biological experiments showing EXO1 catalysing forked, gap and nick DNA substrates with a 5′ flap of (**A**) 1 nt, (**B**) 5 nt and (**C**) 10 nt. (**D**) Statistical analysis of degradation of fork, gap and nick substrate of different length by EXO1. One-way analysis of variance with Tukey's multiple-comparison test with significance. The letters indicate *F*=fork, *G*=gap and *N*=nick substrate, ns=not significant. Stars in the table indicated the significance (*P*<0.05) between the different parameters.

### Full-length EXO1 requires a larger DNA platform

Previously, it was shown, that truncated EXO1 (395 amino acids) cannot process substrates shorter than 6 bp [9]. Full-length EXO1 (846 amino acids) is significantly larger than truncated EXO1 and it is, therefore, possible that full-length EXO1 has different substrate specificity and can process substrates longer than 6 bp. We assayed a 3′ label 42-mer dsDNA (GK142/GK143) substrate in absence and presence of full-length EXO1. The result of this reaction is a 12 bp product suggesting that full-length EXO1 is active on the longer 42-mer dsDNA (Figure 3) and (Figure 4B, lanes 8–10).

### EXO1 has a modest flap activity

Since yeast EXO1 has been shown to process a weak flap activity we assayed this activity of full-length EXO1 on the previous used flap DNA substrate GK145/GK135 (also named A6/A9) [9]. Therefore, we hypothesized that EXO1 processes the GK145/GK135 DNA substrate from the dsDNA end as well as from the 5′ single-stranded DNA end (Figure 4A). To verify this, we used the GK145/GK135 substrate to more fully characterize the full-length EXO1 5′ flap activity (Figure 4B, lanes 2–4) [9]. We investigated if EXO1 flap activity could be altered by shortening the duplex region of oligo GK145/GK135 (21 bp) but did not measure any difference in EXO1 flap activity, when introducing a shorter duplex region of 16 bp (versus 21 bp duplex) (Figure 4B, lanes 5–7). However, at a 12 bp duplex region the flap activity was strongly reduced (Figure 4B, lanes 8–10) and in the case of a 8 bp duplex region, no flap activity was observed (Figure 4B, lanes 11–13) [9].

To confirm that EXO1 processes the 5′ flap substrate from the dsDNA end, we blocked the 5′-dsDNA end with biotin (Figure 4C). In addition, we tested the 5′-biotin substrate after addition of streptavidin, which forms a strong complex with the 5′-biotin, so that EXO1 would not be able to pass and process the dsDNA end [7]. In both assays (blocking 5′-dsDNA end by biotin or biotin-streptavidin) a reduced flap product was observed (Figure 4C, lanes 4–6 and 7–9, respectively). To verify that EXO1 is not bypassing the 5′ biotin or biotin streptavidin, we designed a hairpin with closed dsDNA ends and compared this to the previous flap DNA substrate both with and without blocking the 5′ dsDNA end with biotin. We find that EXO1 has indeed a modest 5′ flap activity compare to flap substrates used in Figures 4(B) and 4(C) (Figure 4D, lanes 8–10).

From the assays in Figures 4(B)–4(D), we conclude that EXO1 can access the substrate from the 5′ single-stranded DNA flap as well from the 5′ dsDNA (Figure 4A). Interestingly, shorter duplexes (12 and 8 bp) (Figure 4B) showed reduced flap activity. This supports the premise that full-length EXO1 cannot process dsDNA shorter than 12 bp dsDNA (Figure 3).

Above we showed (Figures 2A–2F) that temperature modulates EXO1 activity. To assay if lower temperatures influence the endo or flap activity of EXO1, we tested a flap substrate (GK232) at 25°C, 30°C, and 37°C (Supplementary Figure S3A and S3B). At 25°C and 30°C, EXO1 showed an increase in processing the DNA substrate compared with 37°C. When we compared the specific flap activity of EXO1 at 37°C to the flap activity at 25°C and 30°C, we could only detect one product at 37°C, while the lower temperatures resulted in two different products and increased degradation of substrate.

### Cleavage of small flap substrates

It has been shown that FEN1 cleaves 1 nt downstream from the junction at the dsDNA of the 5′ forked flap substrate [38]. EXO1 has been suggested to back up FEN1 during replication [18,19]; therefore we sought to determine the exact position of cleavage of a flap substrate. In Supplementary Figure S3, we show that EXO1 cleaves the substrate into two different products of 8 and 10 nt, respectively, as well as an additional 1 nt product. Therefore, we chose to use a substrate containing a 10 nt 5′ flap of a DNA forked flap (GK247) to verify that the two 8 and 10 nt (flap) products are not specific for the GK232 substrate. The reaction was set up in absence or presence of EXO1 and incubated for 30 min at 37°C. We incubated (Figure 5A) the DNA substrate GK247 in the absence or presence of EXO1 (Figure 5A, lanes 1 and 2) and 10 nt dsDNA oligo (GK245) (lane 3). Our results show that EXO1 flap activity on the 10 nt overhang substrate results in three products: an 11 nt, a weak 9 nt flap and 1 nt product. Our results suggest that EXO1 cleaves 1 nt downstream from the junction at the dsDNA of the 5′ forked flap substrate (11 nt product) and that EXO1 also cleaves to generate an 9 nt flap product and a small product of 1 nt, in the same substrate (Figures 5A and 5B). It cannot be excluded that the two flap products (9 and 11 nt) are a result of DNA thermo-breathing on the structure as we observed above (Supplementary Figure S3A).

FEN1 is essential in the removal of Okazaki fragments at the lagging strand in the process of strand displacement during replication [10]. *In vitro* experiments suggest that 5′ flaps (<5 nt) generated by Pol δ during replication process are efficiently removed by FEN1 [22]. Longer 5′ flaps (>20–30 nt) are removed by FEN1 in collaboration with DNA2 [20]. Therefore, we investigated how 5′ flap substrates with different flap lengths are hydrolysed by EXO1. We show that EXO1 has modest activity in removal of 5′ flaps at DNA fork substrates (Figures 2E and 5A). Interestingly, we show that short flaps (1 nt and 2 nt) are very efficiently processed by EXO1 (Figures 5C, lanes 1–4, 5D and 5E). Interestingly, the forked substrates (1 nt and 2 nt overhang) were found to give a small 5′ flap product (Figures 5C and 5D), in addition to the expected 1 nt product. Small 5′ flaps from the 3 nt overhang up to the 9 nt showed a decrease in total degradation product compared with the 1 and 2 nt flap substrates. Interestingly, within this degraded product, the flap product increases and the 1 nt product decreases, both by increasing length of a 5′ flap (Figures 5C, lanes 5–12, 5D and 5E). The 20 nt flap showed an increased EXO1 activity on the substrate compared with flap substrates of between 3 nt and 9 nt (Figures 5C, lanes 13–14, 5D and 5E). Consistent with Figures 5(A) and 5(B) we observe that the EXO1 main flap product is cleaved 1 bp downstream into the dsDNA. These results suggest possible different routes of 5′ overhang processing between the short and long flaps. Short flap of 1 and 2 nt are efficiently recognized, possibly stimulated by a thermo-breathing effect on the DNA and possibly recognized by tracking. As previously suggested, short flaps up to 5 nt are processed by tracking [39]. However, we show that short flaps between >3 nt and <5 nt are less efficient processed possibly by a less efficient fit in the enzyme. From the crystal structure it was predicted that flap substrates are processed by recognizing first a nick before processing, however the forked substrates are absent of a nick suggesting processing short flaps by tracking [8]. Longer substrates 9 nt and 20 nt both showed an increase in flap and 1 nt products. It is possible that EXO1 can process both routes via tracking and threading. Therefore, it would be interested to design substrates containing structures that are not accessible by threading.

During replication, strand displacement at the lagging strand is essential for cell survival and, therefore, has multiple back up enzymes are advantageous for cell survival. The 5′ flaps activity of EXO1 is suggested to back up FEN1 during replication [18,19]. In support of these data, our biochemical results suggest that EXO1 can play a role in strand displacement in human cells.

### A model for 5′ flap interaction ‘Threading’ or ‘Tracking’

It is suggested that 5′ flap overhangs, shorter than 5 nt, are not subjected to a threading process during replication but rather processed by tracking [21]. In threading the enzyme recognizes the 5′ flap and the processing is mediated by passing the flap through a fully enclosed hole in the protein [40]. The enzyme threads along the ssDNA to the junction with dsDNA and hydrolyse the flap. From the solved crystal structure, it was predicted that EXO1 requires binding at nicks first and then process the 5′ flap, thus ruling out the tracking or threading model (Figures 6C and 6D) [8]. Gloor et al. [20] showed that FEN1 can process short overhangs efficiently by tracking; in case of long flaps (>20 nt), FEN1 requires assistance of DNA2 and is mainly regulated by threading. We show that EXO1 processes short flaps of 1 and 2 nt very efficiently probably removed by tracking (Figures 5B and 5C), and that longer flap (>2 nt) structures are less efficient degraded (Figures 5A and 5C). It has been reported that EXO1 exhibits a flap and 1 nt product on 5′ flap substrates, which suggests that EXO1 possibly recognize a 5′ flap from the ssDNA end [9]. Therefore, we designed three DNA substrates with a 5′ flap of 20 nt to test if threading can occur. The overhangs are blocked for threading by a hairpin formation 6 nt downstream of the 5′ end in the ssDNA region (Figure 6A, lanes 3–4 and 6D). It is possible that this hairpin-mediated blockage can be easily bypassed since such hairpins occur naturally in ssDNA (in the presence of other bases in ssDNA, e.g. C, G and A). To make a stronger blockage, we integrated a modified thymidine residue with biotin–TEG (at position nt 6, from the 5′ ssDNA), which increases the oligo–biotin distance to 15 atoms by using a triethyleneglycol (TEG) spacer. In the presence of streptavidin, the oligo (GK254) containing the TEG-biotin forms a strong noncovalent biological interaction, which would block the EXO1 from sliding over the ssDNA (Figure 6A, lanes 5–6 and lanes 7–8 in presence of streptavidin). Our results suggest that the main process by which EXO1 regulates the endo activity is via tracking, whereas the process of threading contributes minor after the 5′ blocking at the ssDNA to hydrolysing the 5′ flap of 20 nt (Figures 6C and 6D).

### EXO1 processes forked, gap and nick substrates differently

EXO1 has been associated with DNA resection and DNA replication. Above (Figure 5), we observed that EXO1 has a potential role in DNA strand displacement on forked substrates. Therefore, we investigated DNA strand displacement activity on DNA substrates, which represent DNA replication-like intermediates, such as a gap and especially a nicked substrate. The forked, gap and nick substrates can require different actions of EXO1. By exposing EXO1 to three different DNA substrates, forked, gap and nick and to varying lengths of 5′ flap overhang, 1 nt, 5 nt and 10 nt, we observed that EXO1 processes the DNA configurations in different ways. At the 1 nt overhang, only the forked substrate showed two products, 1 nt and an 2 nt flap. The gap and nicked substrate showed only a 1 nt product (Figure 7A). On the forked substrate, the 5 nt flap product, showed three products, 2 flap of 4 and 6 nt, as above described, and additionally a 1 nt product. The gap substrate showed a 5 nt flap and 1 nt product. The nicked substrate only showed a 6 nt flap and 1 nt product (Figure 7B). The 10 nt flap substrates, showed at the forked substrate an 9, 11 nt flap and 1 nt product. The gap substrate showed a 10 nt flap and 1 nt product and the nick substrate showed 11 nt flap and 1 nt product (Figure 5C). Comparing the three different substrates in degradation efficiency, we found no difference in the case of the 1 nt overhang, however, as shown above, the 1 nt overhang is degraded significantly more efficiently compared with the 5 and 10 nt overhang substrates (Figures 5C and 7D). The gap and nick substrate showed no flap product at the 1 nt overhang substrate (Figure 7A, lanes 3–4). Interestingly, the gap substrate cleaves exactly on the DNA junction of the DNA fork, resulting in 5 nt or 10 nt product (Figure 7B, lanes 3–4 and 7C, lanes 3–4). The nicked substrates showed at 5 nt and 10 nt 5′ flap that EXO1 cleaves 1 bp downstream of the dsDNA, akin to the forked substrates, but only has one product at 1 nt, 5 nt and 10 nt overhang substrates (Figure 7A, lanes 6 and 9) The 1 nt overhang forked, gap and nick substrate showed a significant increase in cleavage of the substrate compared with a 5 nt and 10 nt fork, gap or nick substrate. In general, 5 nt or 10 nt forked substrates showed a positive trend in degradation of the substrate. There is no significant difference between the gap and nick substrates on the 5 nt and 10 nt flap substrates (Figures 7A–7D). Interestingly, we confirm strand displacement activity on nicked substrate, which above were detected on a forked substrate (Figure 5A). Nicked substrates are a less likely prone to thermo-breathing of the DNA than the forked substrates. Furthermore, Figure 7 confirms our observation in Figures 5 and 6 that EXO1 is flexible in processing of different flap configurations. EXO1 can possibly process 5′ flaps by tracking, threading and non-threading mediated by either DNA configuration or by conformational change of the EXO1 enzyme [8]. However, these experiments give no direct insight in the mechanism of processing the different flaps substrates. EXO1 is not very efficient in processing flap substrates and, therefore, most probably cooperate with other proteins involved in the process of these types of replication intermediates, such as RPA, binding to ssDNA overhangs or the involvement of helicases for example BLM or WRN.

## CONCLUSION

We provide novel insight into the thermodynamic stability of EXO1. Our results suggest that EXO1 has a strong activity in processing dsDNA and a moderate flap activity, indicating that the EXO1 enzyme differently regulates the two processes. This confirms a role for EXO1 in MMR and double strand break repair where the exonuclease activity is regulated by different enzymes such as MutSα, PCNA, 14-3-3, and 9-1-1 complex [11,14–16] as well as other DNA repair enzymes such as nucleases CtIP and MRN or the RecQ helicases, to resect eroded DNA ends [17,24,25,29–31].

In a recent crystallography study on the HEX-N2, Orans et al. [8] did not observe the 5′ flap activity directly, but their structure indicated that such a single-stranded segment needs to be guided out of the crowded active site [8]. In our study, we re-evaluated the flap activity on different DNA configurations and conclude that EXO1 has a modest 5′ flap activity on a hairpin-flap structure. Additionally, we show novel mechanistic insights into the processing of flap structures, where EXO1 cleaves preferably 1 nt inwards and not at the junction at forked and nicked DNA substrates. Our data support the notion that EXO1 flap activity can function as a backup of FEN1 in strand displacement during replication [22,41]. Our results provide an important contribution to biochemical studies of the EXO1 enzyme in mechanisms of maintaining genome stability in processes of DNA repair and replication.

## Abbreviations

EXO1: exonuclease 1
MMR: mismatch repair
TdT: terminaldexoytransferase
TEG: triethyleneglycol
